# Prejudicial findings regarding suitability for intended purpose during pre‐purchase examinations in a mixed horse population—A retrospective observational study in the United Kingdom

**DOI:** 10.1111/evj.14061

**Published:** 2024-01-17

**Authors:** Annabel V. Shelton, Jason Tupper, David M. Bolt

**Affiliations:** ^1^ Equine Referral Hospital, Royal Veterinary College University of London Hatfield UK

**Keywords:** diagnostic imaging, horse, lameness, pre‐purchase

## Abstract

**Background:**

Pre‐purchase examinations (PPEs) are performed for prospective purchasers of horses to identify prejudicial findings that could make animals unsuitable for an intended use. Although this examination process is often standardised, PPEs remain, in large parts, a subjective procedure. In the United Kingdom, PPEs generally consist of either a two stage (two stage vetting [2SV], i.e., general physical examination at rest and basic trot in‐hand) or a five stage‐examination (five stage vetting [5SV], i.e., general physical exam at rest and after exercise, lameness evaluation including strenuous exercise with re‐evaluation after a period of recovery).

**Objectives:**

To identify the proportion of PPEs with prejudicial findings in a mixed horse population in the United Kingdom and to determine those findings.

**Study design:**

Retrospective observational study.

**Methods:**

PPE certificates from three first opinion equine practices were reviewed. Data collected included practice identity, examination format (i.e., 2SV or 5SV), PPE outcome, radiographs obtained (yes/no), purchase price, animal signalment, intended use and prejudicial PPE findings, if indicated. Prejudicial findings were grouped in 10 subcategories. Descriptive statistics were obtained for all parameters. Non‐normally distributed numeric data between groups were compared using Wilcoxon rank‐sum test. Categorical data were compared between groups using Pearson's chi‐squared test or Fisher's exact test.

**Results:**

Overall, 133 pre‐purchase examination certificates were analysed. Seventy‐six horses (57.1%) had prejudicial findings: Sixty‐one (68.5%) horses undergoing 5SV and 15 (34.1%) of horses undergoing 2SV. Most horses had lameness as the primary prejudicial finding. Horses with a higher purchase price were more likely to undergo 5SV, undergo pre‐purchase radiography, and were more likely to have prejudicial findings identified.

**Main limitations:**

Data were obtained retrospectively from PPE certificates from a single mixed horse population and different results may have been obtained by analysing a larger number of animals or PPEs of horses intended for different uses, from different geographical regions or undergoing a different PPE format. The level of competition in the intended discipline for horses was not recorded. Analysed data were limited to the information recorded on the PPE certificates, and the original radiographs, ultrasonography images and other additional diagnostic data were not reviewed.

**Conclusions:**

Lameness was the most common prejudicial PPE finding. More expensive horses were more likely to undergo a 5SV, have pre‐purchase radiographs obtained and have prejudicial findings identified. These results may help inform prospective studies examining the merits of 5SV versus 2SV formats and the value of inclusion of additional diagnostic imaging in PPEs in the general horse population.

## 1 INTRODUCTION

Pre‐purchase examinations (PPEs) are performed at the request of a prospective purchaser of a horse to identify prejudicial findings that could make the animal unsuitable for an intended use.^1^ PPEs are complex procedures that provide not only clinical, but also logistical and communication challenges for the examining veterinary surgeon undertaking them.^2^ Failure of the examining veterinary surgeon to accurately interpret and report prejudicial findings or to declare conflicts of interest before examining a horse can result in litigation.^3^ This results in considerable pressure for examiners, particularly if they are less experienced. Equine veterinary practice has a long‐standing reputation for being the most litigious discipline of veterinary medicine. Although the PPE process has largely remained unchanged over the past few decades, customer's expectations with regards to guarantees of outcome and speed of reporting appear to have changed.^2^ While comparatively few complaints related to equine work are submitted to the Royal College of Veterinary Surgeons (RCVS), problems associated with PPEs account for approximately one third of them.^2^, ^3^ Approximately two thirds of equine claims to the Veterinary Defence Society (VDS) arise from PPEs.^3^

In the United Kingdom, the British Equine Veterinary Association and the RCVS have provided joint guidance on the examination of a horse on behalf of a prospective purchaser.^4^ The VDS provides a number of additional PPE resources on its website.^5^ The examining veterinary surgeon has, in the first instance, to decide if he/she is able to perform the PPE or must decline due to a conflict of interest, for example if the seller is one of his/her clients.^3^ In the United Kingdom, a standardised pre‐purchase examination is conducted in five stages (five stage vetting, 5SV)^4^: The first stage consists of an external examination of the animal at rest to detect clinically apparent signs of injury, disease or physical abnormalities. This stage includes examination of incisor teeth, the eyes in a darkened area, and auscultation of heart and lungs. The second stage is an examination for soundness: The animal is walked and trotted in hand on firm, level ground. Flexion tests and examination at trot in a circle are not mandatory parts of this stage, but can provide useful additional information. In the third stage, the horse is exercised sufficiently to allow for assessment with increased breathing effort and heart rate. Repeat assessment of the horse's gait at walk, trot and canter is performed. If the horse is not examined under saddle during the third stage, it can also be assessed at the lunge. In the fourth stage, the horse is allowed to rest after exercise and respiratory and cardiovascular system parameters are monitored while they return to baseline levels. The fifth and final stage is a repeat trot‐up in hand to assess for soundness.^4^ Where it is not possible or appropriate to complete all five stages of the PPE or at the specific request of the purchaser, examination can be limited to the first two stages (two stage vetting, 2SV).

The PPE certificate issued to the prospective purchasers should report clinical findings of the examination including those from additional examination procedures, such as detailed oral examination, pre‐purchase radiographs, ultrasonography, results from blood samples or upper airway endoscopy. The certificate should ultimately express the examiner's opinion if the findings on this occasion prejudice a horse's suitability for the intended use.^2^, ^3^, ^4^, ^5^

The aim of the current study was to identify the proportion of PPEs with prejudicial findings regarding suitability of animals for their intended use and to determine these findings in a non‐racing horse population in the United Kingdom. We hypothesised that: (1) lameness is the most common prejudicial finding in PPEs; (2) the frequency of lameness as a prejudicial finding is higher in more expensive horses; (3) the purchase price in horses undergoing 5SV is higher than those undergoing 2SV.

## 2 MATERIALS AND METHODS

### 2.1 Case selection

Clinical records from three first opinion equine practices were reviewed and all PPE certificates from 2021 to 2022 were retrieved. Thoroughbred horses intended for racing were excluded from the study due to absence of PPE certificates.

### 2.2 Parameters

Data obtained from PPE certificates included: Practice identity, PPE format (2SV or 5SV), PPE outcome, radiographs obtained (yes/no), ultrasonographic examination performed (yes/no), purchase price, animal breed, age, sex, intended use and prejudicial PPE findings identified, if indicated. Prejudicial findings regarding suitability for the intended use were categorised into lameness, palpation findings, skin conditions, diagnostic imaging findings, cardiac auscultation findings, respiratory system findings, ocular exam findings, gastrointestinal system findings and unknown. Horse breeds were grouped into Draught Horses, Warmbloods, Light Horses, Ponies or Sports Horses. Intended use of the horse was grouped into Allrounder, Showjumping, Eventing, Dressage, Polo, Hacking or Breeding. Original radiographs, ultrasonographic images and other additional diagnostic data were not reviewed. The reported imaging findings are therefore limited to those recorded on the PPE certificates.

### 2.3 Data analysis

Data for each case was collected on a Microsoft Excel data sheet. A free programming language software (R project for statistical computing, version 4.1.3, r-project.org) was used for analysis. Descriptive statistics were obtained for all parameters. Numeric data were tested for normal distribution using histograms and the Shapiro–Wilk test. Normally distributed numeric data were expressed as mean ± standard deviation and non‐normally distributed numeric data were expressed as mean (range). Non‐normally distributed numeric data between groups were compared using the Wilcoxon rank‐sum test. Categorical data were compared between groups using Pearson's chi‐squared test with Yates continuity correction or Fisher's exact test. Post hoc analysis was performed with pairwise Fisher's exact test using Bonferroni adjustment, if indicated. Statistical significance was set at *p* < 0.05.

## 3 RESULTS

### 3.1 Descriptive analysis

Two‐hundred‐and‐sixteen PPE examinations from the three participating veterinary practices were reviewed. Eighty‐three racing Thoroughbred horses were excluded due to absence of a PPE certificate, which resulted in a final sample size of 133 PPEs. Practice A contributed 58 (43.6%), Practice B 46 (34.6%) and Practice C 29 (21.8%) cases. There were 69 geldings (51.9%), 58 mares (43.6%) and 6 stallions (4.5%). Median horse age was 8 years (range: 16.5 years). Horse type consisted of 41 ponies (30.8%), 33 Sports Horses (24.8%), 24 Light Horses (18.0%), 23 Warmbloods (17.3%) and 12 Draught Horses (9.0%). The intended use indicated on PPE certificates was 67 Allrounder (50.4%), 27 Show jumping (20.3%), 20 Eventing (15.5%), 6 Polo (4.5%), 6 Hacking (4.5%), 6 Dressage (4.5%) and 1 Breeding (0.8%). Eighty‐nine (66.9%) of horses underwent a 5SV and 44 (33.1%) horses underwent 2SV. Radiographs were obtained during the PPE in 37 horses (27.8%). The purchase price could be retrieved for 118 horses (88.7%). The median purchase price was £7000 (range: 1200–55 000). From these, there were 58 horses (49.2%) in price category A (below £7000) and 60 horses (50.8%) in price category B (£7000 and above).

Overall, 76 horses (57.1%) had prejudicial findings regarding their suitability identified during their PPE (Table 1). Primary prejudicial findings included lameness in 42 horses (55.3%), diagnostic imaging findings in 11 horses (14.5%), respiratory system findings in 5 horses (6.6%), skin conditions in 4 horses (5.3%), palpation findings in 3 horses (3.9%), ocular abnormalities in 3 horses (3.9%), cardiac abnormalities in 3 horses (3.9%), one horse (1.3%) each with an identification problem and a gastrointestinal system problem. For three horses (3.9%), the prejudicial finding regarding their suitability for the intended use was not stated (Table 2).

**TABLE 1:** Number, median age, median purchase price, PPE format and radiographs obtained (yes/no) of horses with and without prejudicial findings regarding their suitability for the intended use during PPE.

PPE outcome	Number of horses (%)	Median age (range)	Median purchase price (range)	PPE format		Radiographs obtained	
				2‐stage (%)	5‐stage (%)	Yes (%)	No (%)
No prejudicial findings	57 (42.9%)	8.0 (1.5–18)	£5350 (2000–40 000)	29 (65.9%)	28 (31.4%)	12 (32.4%)	45 (46.9%)
Prejudicial findings	76 (57.1%)	8.0 (1.5–18)	£9500 (1200–55 000)	15 (34.1%)	61 (68.6%)	25 (67.6%)	51 (53.1%)

**TABLE 2:** Prejudicial findings in 133 PPEs by listed by finding category and price category.

	Primary prejudicial finding (76 horses)			Secondary prejudicial finding (12 horses)		
Prejudicial finding category	Price category			Price category		
	A	B	Not indicated	A	B	Not indicated
Lameness	19 (14.4%)	21 (15.96%)	2 (1.52%)	1 (0.12%)	4 (0.48%)	0 (0%)
Palpation elicited pain	1 (0.76%	2 (1.52%)	0 (0%)	1 (0.12%)	1 (0.12%)	0 (0%)
Respiratory	1 (0.76%	3 (2.28%)	1 (0.76%	0 (0%)	0 (0%)	0 (0%)
Skin	3 (2.28%)	0 (0%)	1 (0.76%	0 (0%)	1 (0.12%)	0 (0%)
Diagnostic imaging	0 (0%)	11 (8.36%)	0 (0%)	0 (0%)	3 (0.36%)	0 (0%)
Cardiac	0 (0%)	3 (2.28%)	0 (0%)	0 (0%)	0 (0%)	0 (0%)
Ocular	2 (1.52%)	1 (0.76%	0 (0%)	0 (0%)	0 (0%)	0 (0%)
Gastrointestinal	1 (0.76%	0 (0%)	0 (0%)	0 (0%)	0 (0%)	0 (0%)
Identification	0 (0%)	0 (0%)	1 (0.76%	0 (0%)	0 (0%)	0 (0%)
Unknown	0 (0%)	2 (1.52%)	1 (0.76%	0 (0%)	0 (0%)	0 (0%)

*Note*: There were 76 horses with prejudicial findings regarding suitability for the intended purpose and 12 of these animals had a secondary finding listed. Percentages refer to the proportions within the primary and secondary prejudicial finding groups, respectively. Horses in price category A had a purchase price of less than £7000 and horses in category B a purchase price of £7000 or more.

In 27 (64.3%) of the 42 horses with lameness as the primary prejudicial failing, this was noticed during stage 2 or 3 of the PPE: In 7 (16.7%) of these, lameness was only visible at the lunge. In the remaining 15 horses (35.7%), it was not indicated during which stage lameness was observed. For 7 horses (16.7%), no detailed information with regards to the lameness observed was provided in the PPE certificate. A subjective lameness grade was listed for only 5 (11.9%) of the 42 horses and ranged from 1/10 to 6/10. A positive response to limb flexion was listed in the PPE certificate for 6 (14.3%) of the 42 lame horses. No information regarding flexion tests was obtained from the certificates of the remaining 36 (85.7%) animals.

Of the 11 horses with diagnostic imaging findings listed as the primary prejudicial finding, 8 (72.7%) underwent radiographic examination and 3 (27.3%) underwent radiographic and ultrasonographic examination: In 4 horses (36.4%), impingement of the dorsal spinous processes in the thoracolumbar spine (DSPI) was noted and in 2 (18.2%) osteoarthritis (without indication of an anatomical localisation) was listed on the PPE certificate. One horse (9.1%) with DSPI also had ultrasonographic abnormalities at the origins of the hindlimb proximal suspensory ligaments. Of the other 2 horses undergoing ultrasonographic examination, 1 (33.3%) had a lesion in the deep digital flexor tendon (limb not indicated) and no detailed information about ultrasonographic findings was found on the PPE certificate of the remaining horse. In 5 horses (45.6%), no details with regards to the diagnostic imaging findings limiting the suitability for the intended purpose was provided on the PPE certificate. Of the 5 horses with respiratory system findings listed as primary prejudicial finding, 3 (60%) had left‐sided laryngeal hemiplegia identified on upper airway endoscopy during stage 4 of the PPE, 1 (20%) had an inspiratory stridor at exercise during Stage 3 of the PPE and 1 (20%) presented with unilateral mucopurulent nasal discharge. The 4 horses with skin findings as the primary prejudicial finding during stage 1 of the PPE included 3 animals (75%) with suspected equine sarcoids and 1 horse (25%) with a chronic wound (no localisation indicated). In 3 horses with palpation findings as the primary prejudicial finding during stage 1 of the PPE, 1 animal (33.3%) displayed a pain reaction to palpation of the thoracolumbar spine, 1 animal (33.3%) displayed a pain reaction to palpation of the pelvic musculature and 1 animal (33.3%) had fetlock joint distension in all four limbs. Of the 3 horses with ocular findings as primary prejudicial finding, 1 horse (33.3%) had an immature cataract, 1 horse (33.3%) had bilateral fundic changes and 1 horse (33.3%) was described to have severely impaired vision. All 3 horses with cardiac findings as the primary prejudicial finding had cardiac murmurs at rest identified during stage 1 of the PPE. In the PPE of the horse with an identification problem, the number read with the microchip reader did not match the one in the passport provided. In the horse with gastrointestinal problem as the primary prejudicial finding, there was a concern regarding gastric ulceration, but it was not indicated in the PPE certificate whether the animal underwent gastroscopic examination.

Twelve of the 76 horses (15.8%) with a primary prejudicial finding also had a secondary prejudicial finding listed. This included lameness in 5 horses (41.7%), diagnostic imaging findings in 3 horses (25%), palpation findings in 2 horses (16.7%), and one horse (8.3%) each with a palpation finding and a skin abnormality (Table 2).

### 3.2 Participating practices

Practice A had 37 horses (63.8%), Practice B 25 horses (54.3%) and Practice C 14 horses (48.3%) with prejudicial findings during the study period. The proportion of horses with prejudicial findings was not significant between participating practices, *X* ^2^ (2, *N* = 133) = 2.13, *p* = 0.346.

### 3.3 PPE format and radiographs

Sixty‐one (68.5%) horses undergoing 5SV and 15 (34.1%) of horses undergoing 2SV had prejudicial findings regarding their suitability for the intended use. There was a significantly higher proportion of horses undergoing a 5SV with prejudicial findings, *p* < 0.001. Overall, 37 of 133 (27.8%) horses had radiographs obtained during their PPE: Thirty‐five (94.6%) of horses that had radiographs obtained during their PPE underwent 5SV and two horses (5.4%) underwent 2SV (Table 1). The decision to obtain radiographs was significantly associated with the PPE format (*p* < 0.001, Fisher's exact test). However, horses that had radiographs obtained were not significantly more likely to have prejudicial findings, (*p* = 0.2). Five of 67 intended Allrounders (7.46%), 3 of 6 intended Dressage horses (50%), 12 of 20 intended Eventers (60%) and 17 of 27 intended Showjumpers (62.96%) had radiographs obtained during the PPE. There was a significant difference with regards the decision to obtain radiographs between intended athletic disciplines (*p* < 0.001, Fisher's exact test). Post hoc analysis revealed that Allrounders underwent radiography significantly less frequently compared with horses purchased for Eventing (*p* < 0.001) or Showjumping (*p* < 0.001).

### 3.4 Purchase price

The purchase price was non‐normally distributed: The median purchase price for horses without prejudicial PPE findings was £5350 (range: 2000–40 000), whereas the median purchase price for horses with prejudicial findings was significantly higher £9500 (range: 1200–55 000, *p* < 0.001). Based on the median purchase price, horses were assigned to price category A (below £7000) or price category B (£7000 and above) for further analysis. There was a significantly higher proportion of horses with prejudicial PPE findings in price category B, (*p* = 0.009). Practice A examined 36 horses in price category A (64.3%) and 20 horses in price category B (35.7%), practice B examined 19 horses in price category A (46.3%) and 22 horses in price category B (53.7%), and practice C examined 3 horses in price category A (14.3%) and 18 horses in price category B (85.7%). This difference was significant (*p* < 0.001). Twenty‐seven horses in price category A (46.6%) and 43 horses in price category B (71.7%) had prejudicial PPE findings. Thirty‐one horses (51.7%) in price category A and 2 horses in price category B (3.4%) had radiographs obtained during their PPE (*p* < 0.001). Forty‐seven horses (78.3%) in price category B and 30 horses (51.7%) in price category A underwent 5SV (*p* = 0.004). From the 27 horses with prejudicial PPE findings in price category A, 19 (70.4%) had lameness listed, 3 (11.1%) a skin condition, 2 (7.4%) an ocular condition and 1 (3.7%) each respiratory findings, palpation findings and gastrointestinal system findings. From the 43 horses with prejudicial PPE findings in price category B, two (48.8%) were lame, 11 (25.6%) had diagnostic imaging findings, 3 (7.0%) each had cardiac or respiratory findings, 2 (4.7%) had abnormal palpation findings and one (2.3%) each had subjective, ocular and undeclared findings listed (Table 2). Although overall comparison of the frequency of prejudicial findings was significantly different between the two price categories (*p* = 0.003, Fisher's exact test), post hoc testing failed to identify specific findings that were significantly different between price categories. This was attributed to the uneven distribution of cases in the contingency table, a phenomenon also referred to as Simpson's paradox.^6^

### 3.5 Signalment and intended use

The age difference between horses with or without prejudicial PPE findings was not significant (*p* = 0.8). Horse sex was also not significantly associated with the presence of prejudicial PPE findings (*p* = 0.6, Fisher's exact test). Two of 12 Draught Horses (16.7%), 15 of 24 Light Horses (63.5%), 24 of 41 of Ponies (58.5%), 22 of 33 Sports Horses (66.7%) and 13 of 23 Warmbloods (56.5%) had prejudicial findings regarding their suitability for the intended use. However, these differences in frequency of prejudicial PPE findings between horse types were not statistically significant (*p* = 0.05) Thirty‐seven of 67 intended Allrounders (55.2%), 0 of 1 horses intended for Breeding (0%), 3 of 6 horses intended for Dressage (50.0%), 14 of 20 horses intended for Eventing (70.0%), 3 of 6 horses intended for Hacking (50.0%), 3 of 6 horses intended for Polo (50.0%) and 16 of Horses intended for Showjumping (59.3%) had prejudicial findings. There was no significant association between the intended use and the presence of prejudicial PPE findings (*p* = 0.8, Fisher's exact test). However, when animals were grouped into athletic categories, differences were apparent and 27 of 40 (67.5%) of horses intended for work on a ‘lower’ athletic level (Allrounder, Hacking and Breeding combined) had prejudicial findings, compared with 15 of 36 (41.66%) horses intended for more strenuous athletic use (Showjumping, Eventing, Polo or Dressage combined). This difference was significant (*p* = 0.04).

## 4 DISCUSSION

This study investigated PPE outcomes in a mixed, non‐racing horse population in the United Kingdom. The results confirmed our hypothesis that lameness was the most commonly identified prejudicial finding regarding suitability for an intended purpose. The study further confirmed that horses with a higher purchase price were more likely to undergo a 5SV, to have pre‐purchase radiography, and that they were more likely to have prejudicial PPE findings.

PPEs should not be understood as a binary pass or fail test, but the examination should result in a recommendation based on the opinion of a veterinary surgeon at an isolated point in time.^1^, ^4^ As such, a PPE cannot accurately predict future developments, but it may provide an indication on how the horse will succeed or progress once it is exercising at the intended level. The prospective athletic activity and intended use of a horse makes some components of a PPE more important and can increase the emphasis on examining certain organ systems or specific anatomical areas more closely. This often results in the use of additional diagnostic modalities: Horses purchased for endurance riding or eventing are more likely to undergo upper airway endoscopy during their PPE than, for example, show ponies.^7^ In the current study, we were unable to ascertain if upper airway endoscopy was undertaken routinely in some PPEs or if it was prompted by clinical findings, such as respiratory noise during exercise. Training and competing in different disciplines also put varying demands on various musculoskeletal structures and it is important to ensure that these are healthy during the PPE.^8^ Abnormal visual and palpatory findings in flexor tendons and suspensory ligaments of prospective Sport horses, for example, should prompt ultrasonographic examination.^7^ In younger horses, emphasis should be placed on joints, in particular stifles, hocks, and fetlocks. It is recommended that these joints are examined radiographically for the presence of developmental disorders, such as osteochondritis dissecans or subchondral bone cysts during a PPE.^9^ Consequently, the extent of a PPE can range from a simple physical and lameness examination to a complex assessment with inclusion of additional diagnostic modalities, such as radiography, ultrasonography, upper airway endoscopy or examination of the reproductive tract.^1^, ^2^, ^7^

Lameness was the most common primary prejudicial finding identified in our study. It was listed in 42 (55.3%) out of the total of 76 horses with prejudicial findings. This proportion is in agreement with the findings in a previous study where 52.8% of horses were lame.^1^ However, in the study of van Hoogmoed et al.,^1^ it was not explicitly stated whether identification of lameness resulted in recommending against the purchase of the animal. In horses intended for some athletic disciplines, mild lameness is occasionally tolerated: Western Performance horses used for team roping are often regarded as suitable to perform competitively, even if they are visibly lame. Conversely, lame horses are considered less likely to perform in more strenuous Western disciplines, such as barrel racing.^10^

Age did not have an influence on PPE outcome in our study. An explanation for this could be that mild gait asymmetries or age‐related radiographic findings, such as mild osteoarthritis, are more often tolerated by examiners in older horses intended for low‐level athletic use. However, we were not able to substantiate this with the results of our study.

It has been demonstrated that horses competing in different disciplines are predisposed to injuries at specific anatomical locations and that the type and site of injury may reflect their level of performance.^8^ In agreement with previous reports,^1^, ^11^ our findings have confirmed that a more thorough clinical examination (5SV) and pre‐purchase radiographs were more commonly performed during the PPE of more expensive horses that are intended for intensive athletic use, such as Eventing or Showjumping. This was also reflected in the higher purchase price with a significantly higher proportion of horses in price category B (£7000 or above) undergoing a 5SV, having pre‐purchase radiographs obtained and having prejudicial findings.

Pre‐purchase radiographs are known to directly affect both sale outcome and purchase price during PPEs.^1^, ^12^ Purchase of a horse for a more intense athletic use, such as Eventing or Showjumping, and a higher purchase price, may convince both examiner and prospective purchaser to invest in additional diagnostic modalities, such as radiography, ultrasonography, or upper airway endoscopy. However, radiography is not considered an essential part of every PPE.^10^ The modality is considered useful to screen horses for the presence of certain conditions, such as osteochondritis dissecans,^2^ but other radiographic pathologies, such as navicular bone changes, have been shown to be poor prognosticators for future soundness.^1^, ^13^ In their guidelines, the German Equine Veterinary Association (GEVA) lists a selection of standard radiographic views and grades radiographic features with regards to carrying risk of a horse becoming unsound in the future.^14^ Although this information is helpful, radiographs should not replace the PPE process, but merely represent a component of it^15^: It has been reported that horses intended for certain disciplines, such as Showjumping, are occasionally purchased based on examination of radiographs alone.^2^ Amateur purchasers in particular may misunderstand the absence of radiographic pathology as a reliable prognosticator for future soundness and competition success.^16^ This represents a concern, because, without the context of a clinical examination, it is difficult to qualify the relevance of radiographic findings and their prognostic value with regards to a horse's intended use.^1^, ^15^

The task of identifying a suitable, experienced, and non‐biased examiner lies with the prospective purchaser. In the current study, 15 different veterinary surgeons with various experience levels from three different practices were undertaking the PPEs. Whereas this high number of examiners does not necessarily directly affect the conclusions that can be drawn from the data, it highlights the element of subjectivity in the PPE process: There is a high likelihood that different examiners would, for example, disagree between themselves whether a horse is lame or if radiographic features represent prejudicial findings with regards to the suitability for the intended use. PPEs are therefore, in large parts, a subjective assessment and the results of the current study are susceptible to bias as a result. Although experienced veterinary surgeons with discipline‐specific knowledge are usually enrolled in PPE of more expensive horses and/or horses intended for elite athletic use, there is no specific qualification relating to PPEs required in the United Kingdom.

There are limitations in the current study which limit the conclusions that can be drawn from its findings: Data were obtained from a cohort sample of a non‐racing horse population in the United Kingdom with a high proportion of ponies, Sports horses and Warmbloods. Approximately half of the animals were intended for ‘lower’ level athletic use. Considerably different results may have been encountered with a higher number of animals or by analysing PPEs of horses intended for different use, of horses in different geographical regions or of horses undergoing a different format of PPE. Therefore, most findings of this study likely only apply for the population examined. We were also not able to retrieve the level of competition within the intended discipline for which the horse was examined. Athletic requirements between elite and non‐elite competing horses in Eventing differ considerably.^8^ Horses examined with the prospect of performing at non‐elite level could therefore potentially have been perceived as having dissimilar requirements for passing the PPE by some of the examiners.

The raw images from radiography, ultrasonography and endoscopy were not reviewed for this study and our data relied entirely on the information contained within the PPE certificates. Important information, such as the stage at which a prejudicial finding was identified, lameness grades, details of diagnostic imaging findings was missing on many PPE certificates. This lack of detail is disappointing and has limited the conclusions that can be drawn from the study: For example, horses being considered sound during stage 2, but lame during stages 3 or 5 of the PPE, would strongly support performing 5SV in preference to the 2SV PPE format. Unfortunately, due to missing data, this could not be assessed.

The PPE format was determined in all cases after discussion with the prospective purchaser prior to the assessment. The majority of horses that had lameness as the primary prejudicial finding underwent a 5SV: In 84.4% of these animals, lameness was detected during stage 2 or 3 of the PPE: It appears unlikely that horses that presented lame during the early stages of the PPE subsequently completed the remaining stages of the exam. Unfortunately, the exact proportion of aborted 5SV could not be retrieved from our records and it is likely that an unknown proportion of these exams were in fact limited to the first two stages.

In conclusion, the results of this study support the hypotheses that lameness is the most common prejudicial PPE finding with regards to intended use and that more expensive horse are more likely to undergo a 5SV, have pre‐purchase radiographs obtained, and have prejudicial PPE findings. However, an important limitation of our retrospective study design meant that it was not possible to determine in which of the five stages these prejudicial findings were identified. Stronger conclusions could likely be drawn with a prospective study design, a larger sample size, by involving more practices and examiners and by including horses from more different disciplines.

## AUTHOR CONTRIBUTIONS

**Annabel Shelton:** Data curation; investigation; methodology; writing – original draft. **Jason Tupper:** Conceptualization; methodology; supervision; writing – original draft. **David M. Bolt:** Conceptualization; data curation; formal analysis; investigation; methodology; project administration; validation; writing – original draft; writing – review and editing.

## FUNDING INFORMATION

There was no external or internal funding for this study.

## CONFLICT OF INTEREST STATEMENT

The authors declare no conflicts of interest.

### PEER REVIEW

The peer review history for this article is available at https://www.webofscience.com/api/gateway/wos/peer-review/10.1111/evj.14061.

## DATA INTEGRITY STATEMENT

David M. Bolt had full access to all data and takes responsibility for data integrity and analysis.

## ETHICAL ANIMAL RESEARCH

Research ethics committee oversight not required by this journal: Retrospective study of clinical records.

## INFORMED CONSENT

Explicit owner consent for animals' inclusion in the study was not stated.

## Data Availability

Data supporting the findings of this study are available from the corresponding author upon reasonable request. Data sharing exemption is granted by the editor for this retrospective case series.
